# Quality of oil extracted by cold press from *Nigella sativa* seeds conditioned and pre‐treated by microwave

**DOI:** 10.1002/fsn3.4021

**Published:** 2024-02-09

**Authors:** Mina Sanati Agah, Sodeif Azadmard‐Damirchi, Samad Bodbodak

**Affiliations:** ^1^ Department of Food Science and Technology, Faculty of Agriculture University of Tabriz Tabriz Iran

**Keywords:** extraction, oil yield, oxidative stability, seed, vegetable oil

## Abstract

Black cumin (*Nigella sativa*) seed (BS) oil has received much interest in the food and pharmaceutical industries due to its valuable nutritional properties, but this oil has low oxidative stability. The effect of microwave pre‐treatment at 0 to 2.5 min and conditioning with different buffers at pH 3 to 9 of BS, before oil extraction by cold press, were investigated. The oil extraction yield was higher; acid value (AV) and peroxide value (PV) were lower in the oil extracted from seeds, which were first microwaved and then moisturized and vice versa. BS with pH 3, microwave time of 1.25 min, and moisturizing level of 5%, which gave oil extraction yield of 27.2%, AV of (2.9 mg NaOH/g oil), and PV of (8.3 meq O2/kg oil), was selected as an optimum sample and its quality changes were investigated during storage compared with the oil extracted from the control sample (without any pre‐treatment). In conclusion, the oil extracted from the pre‐treated BS had higher bioactive components and lower AV and PV during the storage; therefore, microwave radiation and pH adjustment before oil extraction from BS by cold press are recommended.

## Graphical abstract

Black cumin (*Nigella sativa*) seed (BS) oil has got much interest in the food and pharmaceutical industries due to its valuable nutritional properties, but this oil has low oxidative stability. The effect of microwave pre‐treatment and conditioning with different buffers, before oil extraction by cold press were investigated. It was approved that the oil extracted from the pre‐treated BS had higher bioactive components and lower acid value (AV), and peroxide value (PV) during the storage; therefore, microwave radiation and pH adjustment before oil extraction from BS by cold press is recommended.

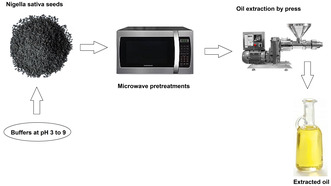

## 1 INTRODUCTION

Black cumin seed (BS) (*Nigella sativa*L.) is an annual plant of the Ranunculaceae family. This plant grows in Western Asia, North Africa, and Southern Europe. BS has many edible, medicinal, and cosmetic uses (Kiralan et al., 2021; Ramadan, 2021). BS can give oil from 25% to 40% depending on the oil extraction method, seed moisture content, seed pre‐treatment type, and conditions (Dezashibi et al., 2022).

Black cumin seed oil can be extracted by press (hot or cold pressing methods) or solvent. Solvent extraction and hot press give higher oil extraction yield, but the obtained oil has to be refined due to higher impurities (Kiralan et al., 2014). Oil extracted by cold press is getting more attention and demand, because it does not require refining processes, and it is free of solvents and chemicals. The cold press BS oil contains many phenolic and health‐enhancing bioactive components, the most important of which is thymoquinone (Alkhatib et al., 2020).

One of the main problems in the production of BS oil is its high peroxide value (PV) and acid value (AV), even on the extraction day (Ashrafi et al., 2023). Various methods have been used to reduce the PV and AV of this oil. Mixing in different ratios with other vegetable oils or pressing a mixture of BS with other oilseeds, such as sunflower seeds, has been used (Mazaheri et al., 2019a; Ramadan & Wahdan, 2012).

It has been reported that enzymes, such as lipase and lipoxygenase in the BS, may reduce the extracted oil quality (Mazaheri et al., 2021). Thermal processing of foods is a well‐established method for inactivating enzymes for prolonging the shelf life and quality of the finished products. However, due to the destructive effects of high temperatures on some quality characteristics, alternative non‐thermal methods, such as microwaves, are used (Mazaheri et al., 2019b). It has been reported that using the microwave to pre‐treat seeds before oil extraction is a simple and appropriate method to obtain oil with high bioactive components such as phenolic compounds and also with longer shelf life (Rękas, Ścibisz, et al., 2017, Rękas, Wroniak, & Ścibisz, 2017). More efficient oil extraction by press can be achieved through microwave irradiation, because the cell membrane is ruptured, which makes oil extraction easier. Also, it has been demonstrated that using this method for oilseeds can increase the content of tocopherols, phytosterols, and phenolics in the extracted oils (Azadmard‐Damirchi et al., 2010).

Considering the optimum pH of enzyme activity in plant sources, changing the pH of oilseeds can be a method to reduce the activity or inactivation of enzymes and further improve the quality characteristics of the extracted oil (Lampi et al., 2020).

As mentioned above, there have been many efforts to pre‐treat the BS with different methods to extract oil with low PV and AV; however concluding the need for further research to lower the PV and AV with other suitable methods. Therefore, the present study aims to investigate the effect of different buffers to adjust pH with microwave pre‐treatment of BS on the quality properties of extracted oil by cold press.

## 2 MATERIALS AND METHODS

### 2.1 Sample

Black cumin seed (*Nigella sativa*L.) samples were obtained from the local market (Tabriz, Iran). Solvents were obtained from Merck (Darmstadt, Germany).

### 2.2 Microwave pre‐treatment

Five hundred grams of seeds was placed in Pyrex Petri dishes (26‐cm diameter) of the microwave (Model: MW2300 GF, 800 W). Samples were microwave treated at a frequency of 2450 MHz at three times of radiation (0, 1.25, and 2.5 min).

### 2.3 Conditioning

Buffer systems with a pH range of 3–9 included Tris (pH 9), sodium acetate (pH 3), and phosphate buffer (pH 6) with the conditional levels of 0–10 (w/w %) were used to moisturize the BS.

### 2.4 Seed pre‐treatment

There were two options to condition and pre‐treat the BS as follows:

1. Moisturizing, then keeping in refrigerator for 24 (h) and then microwaving, and oil extraction by cold press.
2. Microwaving and then moisturizing, then keeping in refrigerator for 24 (h) and then oil extraction by cold press.

### 2.5 Oil extraction by pressing

Oil was extracted by using a mechanical screw press apparatus (model P500R, Anton Fries, Germany) at below 50°C. Then it was filtered and stored at 4°C for a short time until experiments started.

### 2.6 Oil extraction yield

The oil extraction yield was determined, according to the following Equation 1.

(1) $$\text{Extraction yield}\;\left(\%\right)=\frac{\text{Extracted oil amount}\;\left(g\right)}{\text{Initial seed weight}\;\left(g\right)}\times 100$$

### 2.7 Peroxide value

The peroxide value (milliequivalent of oxygen per kilogram (meq O2/kg of oil samples)) was determined using an American Oil Chemists’ Society (AOCS) (1997) official method (Cd 8–53).

### 2.8 Acid value

The acid value (mg NaOH/g of oil samples) was determined using an AOCS (1997) official method (Cd 5a‐40).

### 2.9 Total phenolic compounds

The total phenolic compounds (TPCs) of the BS oil were determined using the Folin–Ciocalteu method (Womeni et al., 2016). The absorbance of the oil mixture was determined at 760 nm using a UV–Vis spectrophotometer (Cecil Instruments Ltd., Cambridge, UK). The phenolic compound of oil was expressed as gallic acid equivalents (GAEs) (mg gallic acid/kg of oil).

### 2.10 Determination of chlorophyll and carotenoid contents of BS oil

Chlorophylls and carotenoids of BS oil were determined in 670 nm and 470 nm, respectively, by dissolving in cyclohexane (Suri et al., 2020), and contents were estimated using the following Equations 2 and 3.

(2) $$\text{Chlorophylls content}\;\left(\mathrm{mg}/\mathrm{kg}\right)=\left(\;{\mathrm{ABS}}_{670}\times 10^{6}\right)/\left(613\times 100\times \text{density}\right)$$

(3) $$\text{Carotenoids content}\left(\mathrm{mg}/\mathrm{kg}\right)=\left(\;{\mathrm{ABS}}_{470}\times 10^{6}\right)/\left(2000\times 100\times \text{density}\right)$$

where A is the absorbance and L is the spectrophotometer cell thickness (1 cm).

### 2.11 Oxidative stability

The oxidative stability of oil samples was determined by a Rancimat Instrument, according to AOCS (1993) standard method (Cd 12b–92).

### 2.12 Fatty acid composition

The fatty acid composition of oil extracted from the optimum and control (without any pre‐treatment) BS was determined by gas chromatography (GC) using the previously published method (Yahyavi et al., 2020). In brief, fatty acid methyl esters (FAMEs) of the oil samples were prepared through transesterification of the oils using methanolic potassium hydroxide, and then FAMEs analyzed by GC (Agilent 7890 B, Agilent, USA). The GC device featured a flame ionization detector (FID), split/splitless injector, and BPX70 capillary column (50 m × 0.22 mm, 0.25 μm). Helium was used as the carrier gas, and nitrogen was used as the make‐up gas. Temperatures of the injector and detector were set at 230°C and 250°C, respectively. One microliter of FAME dissolved in n‐hexane was injected in split injection mode at a split ratio of 25:1. Temperature of the oven was initially set at 158°C for 5 min and then increased to 220°C and retained for 10 min. Detection of FAMEs and determination of fatty acid composition were performed by comparing the retention time with those of the relevant standards and peak areas used to calculate the fatty acid percentage.

### 2.13 Statistical analysis

A central composite design by response surface methodology was used to determine the BS pre‐treatment conditions using three independent variables (pH (3–9), soaking level (0%–10%), and microwave time (0–2.5 min)). The ranges and the center points for the three independent variables were based on the results of preliminary experiments (Table 1). The central composite design in the experimental design consists of 20 runs and 5 replicates of the central point. The analysis of variance (ANOVA) and the coefficient of determination (linear) were used to estimate the models’ desirability. After selecting the OS, further tests were performed by SPSS software (IBM SPSS Statistics 23). The statistical analysis was conducted by one‐way ANOVA, and the mean difference between the data was implemented by the Duncan test at the probable level of 5% (*p* < .05).

**TABLE 1:** Pre‐treatment condition of *Nigella sativa* seeds before oil extraction by cold press.

Run	pH	Microwave time (min)	Moisture content (%)
1	3	2.5	0
2	6	1.25	5
3	6	1.25	5
4	9	2.5	10
5	6	2.5	5
6	6	1.25	0
7	6	0	5
8	3	1.25	5
9	3	0	10
10	6	1.25	5
11	3	2.5	10
12	6	1.25	5
13	6	1.25	10
14	9	0	10
15	9	1.25	5
16	6	1.25	5
17	3	0	0
18	6	1.25	5
19	9	2.5	0
20	9	0	0

## 3 RESULTS AND DISCUSSION

### 3.1 Oil extraction yield

The oil content of seeds is one of the important factors in evaluating their nutritional quality and economic value. Oil extraction yield is affected by various factors, including harvest time, seed type, seed condition (maturation and moisture content), oil extraction method, and seed pre‐treatment (Azadmard‐Damirchi et al., 2010).

According to the results of the oil extraction yield, soaking of seeds after microwave pre‐treatment could give a higher oil extraction yield (from 3% to15%) compared to the treatment of soaking and then microwave (Table 2).

**TABLE 2:** Oil extraction yield (%), acid value (mg NaOH/g oil), and peroxide value (meq O2/kg oil) of oil extracted from pre‐treated black cumin seed.

Run	Oil extraction yield		Peroxide value		Acid value	
	Microwave–soaking	Soaking–microwave	Microwave–soaking	Soaking–microwave	Microwave–soaking	Soaking–microwave
1	30.2^a^ ± 0.1	28.8^b^ ± 0.1	12.1^a^ ± 0.1	12.4^a^ ± 0.1	2.9^a^ ± 0.1	3.0^a^ ± 0.1
2	29.7^a^ ± 0.1	26.0^b^ ± 0.1	14.7^a^ ± 0.1	17.5^b^ ± 0.1	3.5^a^ ± 0.1	4.1^b^ ± 0.1
3	28.8^a^ ± 0.1	24.3^b^ ± 0.1	14.7^a^ ± 0.1	17.3^b^ ± 0.1	3.4^a^ ± 0.1	4.2^b^ ± 0.1
4	24.6^a^ ± 0.1	28.2^b^ ± 0.1	15.8^a^ ± 0.1	18.5^b^ ± 0.1	3.7^a^ ± 0.1	4.6^b^ ± 0.1
5	29.0^a^ ± 0.1	30.5^b^ ± 0.1	11.8^a^ ± 0.1	15.7^b^ ± 0.1	3.4^a^ ± 0.1	4.0^b^ ± 0.1
6	27.4^a^ ± 0.1	26.8^a^ ± 0.1	11.0^a^ ± 0.1	11.6^a^ ± 0.1	3.1^a^ ± 0.1	3.2^a^ ± 0.1
7	24.4^a^ ± 0.1	20.4^b^ ± 0.1	13.4^a^ ± 0.1	13.4^a^ ± 0.1	3.7^a^ ± 0.1	3.9^a^ ± 0.1
8	27.2^a^ ± 0.1	25.5^b^ ± 0.1	6.3^a^ ± 0.1	10.8^b^ ± 0.1	2.9^a^ ± 0.1	3.4^b^ ± 0.1
9	19.4^a^ ± 0.1	20.0^a^ ± 0.1	9.3^a^ ± 0.1	9.7^a^ ± 0.1	3.6^a^ ± 0.1	3.5^a^ ± 0.1
10	27.1^a^ ± 0.1	23.5^b^ ± 0.1	14.5^a^ ± 0.1	17.5^b^ ± 0.1	3.5^a^ ± 0.1	4.1^a^ ± 0.1
11	25.0^a^ ± 0.1	29.7^b^ ± 0.1	7.6^a^ ± 0.1	12.0^b^ ± 0.1	3.1^a^ ± 0.1	3.6^b^ ± 0.1
12	28.6^a^ ± 0.1	26.8^b^ ± 0.1	14.5^a^ ± 0.1	17.6^b^ ± 0.1	3.5^a^ ± 0.1	4.1^a^ ± 0.1
13	26.7^a^ ± 0.1	21.6^b^ ± 0.1	15.3^a^ ± 0.1	18.4^b^ ± 0.1	4.0^a^ ± 0.1	4.6^b^ ± 0.1
14	18.2^a^ ± 0.1	18.5^a^ ± 0.1	15.0^a^ ± 0.1	15.0^a^ ± 0.1	4.9^a^ ± 0.1	5.1^a^ ± 0.1
15	26.1^a^ ± 0.1	26.0^a^ ± 0.1	14.6^a^ ± 0.1	17.7^b^ ± 0.1	4.0^a^ ± 0.1	4.8^b^ ± 0.1
16	27.8^a^ ± 0.1	26.0^b^ ± 0.1	14.5^a^ ± 0.1	17.8^a^ ± 0.1	3.4^a^ ± 0.1	4.1^b^ ± 0.1
17	21.6^a^ ± 0.1	21.5^a^ ± 0.1	11.3^a^ ± 0.1	11.7^a^ ± 0.1	3.6^a^ ± 0.1	3.4^a^ ± 0.1
18	28.1^a^ ± 0.1	27.8^a^ ± 0.1	14.7^a^ ± 0.1	17.2^b^ ± 0.1	3.4^a^ ± 0.1	4.2^b^ ± 0.1
19	29.8^a^ ± 0.1	29.5^a^ ± 0.1	12.3^a^ ± 0.1	12.5^a^ ± 0.1	2.9^a^ ± 0.1	3.1^a^ ± 0.1
20	21.5^a^ ± 0.1	21.6^a^ ± 0.1	11.5^a^ ± 0.1	11.5^a^ ± 0.1	3.6^a^ ± 0.1	3.6^a^ ± 0.1

*Note*: Different superscripts within the same line represent significant difference at (p < 0.05).

Generally, a longer microwave pre‐treatment period (2.5 min) could give a higher oil extraction yield (28.8%–30.5%). However, results showed that a longer microwave period could reduce the oil quality, which will be discussed in the PV and AV section. Also, it was noticed that the radiation period of more than 2.5 min could damage the BS and generate smoke.

The lowest oil extraction yield (18.2%) was related to a sample with pH 9, a soaking level of 10% without a microwave (Table 2). This result shows that pre‐treatments in suitable condition can enhance the oil extraction yield; however, some pre‐treatment conditions can lower the oil extraction yield, which should be kept in mind during the oilseed pre‐treatments and oil extraction process. The obtained results showed that high level of moisture content of seeds can reduce oil extraction yield, probably due to emulsification and also formation of a paste‐like seed cake, which lower the oil separation from the seed cake during pressing. Also, high moisture content can reduce the friction, which reduces the cell rupture, and therefore causes a significant reduction in oil extraction.

Also, the results of the analysis according to the linear model for samples soaked after microwave pre‐treatment showed that microwave radiation period and soaking level had a statistically significant effect on the oil extraction yield (*p* < .05), while the pH had no significant impact (*p* ≥ .05). This is because the microwave pre‐treatment of oilseeds with proper humidification enhances the mass transfer coefficient due to severe rupture of cell membranes and reduces the oil extraction barriers (Azadmard‐Damirchi et al., 2010). Moreover, microwaves at a suitable period could generate stable pores in the seeds, allowing the oil to move as much as possible through their porous cell walls. These results were consistent with the previously published data by Rękas, Ścibisz, et al. (2017), Rękas, Wroniak, & Ścibisz, (2017).

In addition to the microwave pre‐treatment, the moisture content of the oilseed could significantly affect the oil extraction yield. It has been reported that different oilseeds have an optimum moisture level to give a higher oil extraction yield by the pressing method (Siabi et al., 2023). According to the obtained results, generally soaking after microwave pre‐treatment could give a higher oil extraction yield. Also, soaking of BS at a level of 5% could provide a higher oil extraction yield (Table 2). As mentioned above, the high moisture content could reduce the oil extraction yield, also very low moisture content of oil seeds is not suitable as well, as it could generate high friction and heat, which can affect the oil composition. In addition, very low moisture content of oil seeds destroys the screw of press equipment and also lowers the oil extraction yield. Intermediate level of moisture content of oil seeds could generate proper friction and cell rupture and with less emulsification, which provide high oil extraction yield. These results are in agreement with previously published data (Mazaheri et al., 2021).

### 3.2 Peroxide value

Peroxide value is one of the important factors in oil quality evaluation. This value is obtained from the quantitative measurement of hydroperoxides as primary oil oxidation products (Rękas, Ścibisz, et al., 2017; Rękas, Wroniak, & Ścibisz, 2017). Lower PV shows the high quality of the vegetable oils. There is established value for PV by the national and international standards for cold press and refined vegetable oils due to the importance of PV in vegetable oil quality. The maximum PV for cold press vegetable oils established by Codex Alimentarius is 15 (meq O2/kg oil) (Alimentarius, 2019).

For oil extracted from BS soaking of seeds after microwave pre‐treatment, the highest PV (15.8 meq O2/kg oil) was related to the sample of 2.5 min microwave irradiation, pH 9, and 10% soaking level. The lowest PV (6.3 meq O2/kg oil) was related to the sample with microwave time of 1.25 min, pH 3, and 5% soaking level (Table 2). The results of analysis according to the linear model showed that the increasing microwave period prevents PV increment (Table 2). The high moisture content can make condition suitable for enzymatic activity, which causes oil deterioration and PV increment.

For oil extracted from BS soaked and then pre‐treated by microwave, results showed that the highest PV (16.5 meq O2/kg oil) was related to the sample with 2.5 min microwave, pH 9 at 10% soaking level. The lowest PV (5.8 meq O2/kg oil) was also obtained for the treatment of the microwave time of 1.25 min, pH 3, and the conditional level of 5% (Table 2).

The results of the PV experiment on the first day of oil extraction showed that, generally, the PV was lower for oil extracted from BS when the soaking was performed after microwave pre‐treatment. This is probably because the oil oxidizing enzymes lose their activity during microwave pre‐treatment, and therefore this step prevents the PV rise during the soaking and conditioning (Mazaheri et al., 2019b).

Results showed that generally, microwaves alone could not affect the PV of oil extracted from BS (Table 2). Also, pH adjustment showed that an increase in the pH of BS can adversely affect the PV of extracted oils. According to the PV of soaked BS, low and high moisture content could produce oil with high PV. These results are in agreement with the previously published data (Mazaheri et al., 2019b).

In oilseeds, the optimum pH for the activity of oxidation enzymes and lipases is found to be present in the neutral to alkaline range. Reducing the pH to less than the optimum level can cause a decrease in the activity of enzymes (Lampi et al., 2020). Results of this study also showed that lower pH (3) of the moisturizing medium could give oil with lower PV (Table 2).

### 3.3 Acid value

Acid value is one of the important indicators in evaluating oil quality. The AV indicates the amount of free fatty acids in the oil due to the hydrolysis of triacylglycerols. High acidity in oils reduces the smoke point and accelerates their oxidation (Drinić et al., 2020).

The results of AV for the oil extracted from BS pre‐treated by microwave and then soaked showed that the highest AV (4.9 mg NaOH/g of oil) was related to oil extracted from BS treated with the buffer of pH 9 and soaking level of 10% without microwaves, and the lowest AV (2.9 mg NaOH/g of oil) was found for oil extracted from BS treated only with microwaves for 2.5 min. The analysis of results according to the linear model showed that the microwave time, pH, and the soaking level had a significant effect (*p* < .05) on the AV. This is probably because heat generated during the microwave pre‐treatment can reduce the activity of the lipase enzyme and, as a result, affect the free fatty acid formation and reduce the AV.

Various studies showed that the lipase enzyme has optimum activity in a medium with neutral to alkaline pH. By reducing the pH to less than the optimum condition, the activity of the enzymes can be affected and decreased significantly due to changes in the enzyme active site and reduction in the rate of hydrolysis reaction (Lampi et al., 2020).

The obtained results showed that the combination of microwave pre‐treatment and conditioning with the acid buffer of BS could cause a significant reduction in AV of extracted oils. Applying acidic buffer alone in the pre‐treatment of BS did not show a significant difference in AV of extracted oils (Table 2). Generally, increased moisture content to more than 5% also increased the AV. Also, it has been reported that increasing the moisture and microwave at higher level duration in the treatment of BS can increase the AV (Rękas, Ścibisz, et al., 2017; Rękas, Wroniak, & Ścibisz, 2017).

According to the results of the AV tests for oil extracted from BS soaked and then pre‐treated by microwave on the first day of oil extraction, the highest amount of AV (5.1 mg NaOH/g of oil) corresponds to the treatment without microwave, pH 9, and conditional level of 10%, and the lowest value (3 mg NaOH/g of oil) corresponds to the sample with a microwave time of 2.5 min.

According to the obtained results from oil extraction yield, PV, and AV, and also the results of Design Expert, microwave pre‐treatment and then soaking of BS could give the best oil quality and higher oil extraction yield. Also, it was demonstrated that microwave time of 1.25 min and soaking at a 5%, and pH 3 were the optimum conditions for pre‐treatment of BS before oil extraction by cold press.

### 3.4 Changes in the BS oil quality during storage

Oil extracted from the BS at optimum conditions was analyzed for PV, AV, fatty acid composition, and bioactive components and compared with the oil extracted from the control BS (without any pre‐treatment) during the storage for 90 days at room condition (Lampi et al., 2020).

### 3.5 Peroxide value

The obtained results showed that PV was increased in both extracted oil samples (optimum and control), but the increment rate was slow in the oil extracted from BS pre‐treated by the optimum condition (OC) (Table 3). PVs of OC and oil extracted from the control sample were 18 and 33 (meq O2/kg oil), respectively, which show the higher stability of OC during storage. This can be explained by the higher antioxidative properties in OC and the low oxidizing enzyme activity (Lampi et al., 2020).

**TABLE 3:** Acid value (mg NaOH/g oil) and peroxide value (meq O2/kg oil) of oil extracted from the *Nigella sativa* seeds without pre‐treatment (control) and with pre‐treatment in the optimum condition (optimum) during storage.

Experiments	Day 1		Day 30		Day 60		Day 90	
	Optimum*	Control	Optimum	Control	Optimum	Control	Optimum	Control
Acid value	2.9 ± 0.01^a^	3.6 ± 0.01^b^	3.2 ± 0.01^a^	5.1 ± 0.01^b^	4.0 ± 0.01^a^	10.6 ± 0.01^b^	8.4 ± 0.01^a^	15.2 ± 0.01^b^
Peroxide value	6.3 ± 0.01^a^	11.5 ± 0.01^b^	8.2 ± 0.01^a^	12.9 ± 0.01^b^	15.1 ± 0.01^a^	19.1 ± 0.01^b^	18.2 ± 0.01^a^	33.3 ± 0.01^b^

*Note*: Different superscripts within the same line represent significant difference at (*p <* .05).

* pH 3, microwave time 1.25 min, and soaking level 5%.

Peroxide value of the oil extracted by the cold press should not exceed 15 (meq O2/kg oil), as it is a maximum level established by international standards (Codex 1999). Results showed that OC has higher shelf life than oil extracted from the control sample according to the PV data (Table 3). It should also be mentioned that these oils were kept in room condition; if the oils were kept in a better situation, such as lower temperature in a refrigerator, the shelf life would be even longer (Sanmartin et al., 2018).

### 3.6 Acid value

Acid value of OC and control oil increased during storage, but it was lower in OC compared with the control oil sample (Table 3). This is probably because of the inactivation of lipase enzymes during heat generation of microwave radiation (Lampi et al., 2020).

Codex Alimentarius limit for AV of cold press oil is 4 (mg KOH/g oil). AV of the oil extracted from the control sample was higher than the Codex Alimentarius limits on day 30, but for the OC it was higher on day 90 of storage (Table 3). These results show that BS oil is very susceptible to hydrolysis, and there is a need for further research to seek other methods of BS pre‐treatments to control the free fatty acid formation and increase of AV.

### 3.7 Total phenolic compounds (TPCs)

Part of the antioxidative property of BS oil is related to TPC. The variety, oil extraction method, processing, and storage conditions are essential factors in the number of phenolic compounds in vegetable oils (Suri et al., 2020).

**FIGURE 1:**
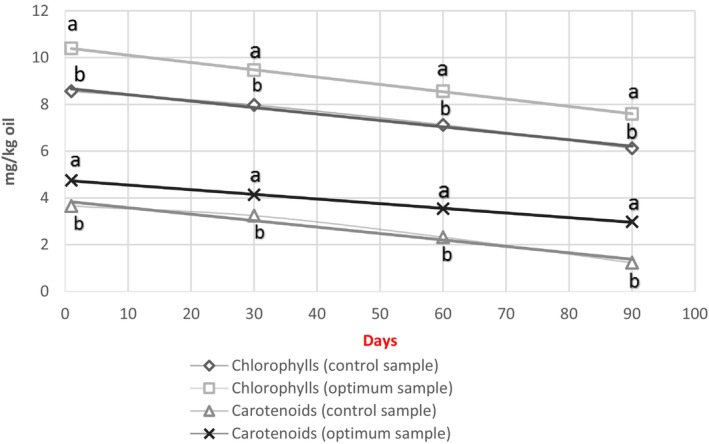
Chlorophyll and carotenoid contents of oil extracted from the *Nigella sativa* seeds without pre‐treatment (control sample) and with pre‐treatment in the optimum condition (optimum sample).

The results showed that microwave pre‐treatment enhances the extraction of TPCs and their migration to the extracted oil due to the cell membrane rupture, denaturation of proteins linked to phenolic compounds, and consequently the more release of TPCs to the extracted oil. Therefore, the OC had a TPC of 262 (mg of gallic acid/kg of oil), which was higher than that of the control sample (185.7 mg of gallic acid/kg of oil) (Figure 1). The amount of TPC of oil samples decreased by 40% due to degradation during storage (Figure 1). These results are following the previously published data on canola and almonds (Lin et al., 2016; Rękas, Ścibisz, et al., 2017; Rękas, Wroniak, & Ścibisz, 2017). Also, Suri et al. (2022) observed that the highest TPC (208.4 μg GAE/mL) was recorded in the oil of BS heated at 720 W for 10 min. The higher phenolic compounds in OS can explain the higher stability and lower PV of this oil during storage compared to the control oil sample.

### 3.8 Determination of chlorophyll and carotenoid

Chlorophyll and carotenoids are natural pigments found in vegetable oils. These pigments are very important in oil quality, oxidative stability, and consumer acceptance (Rękas, Ścibisz, et al., 2017; Rękas, Wroniak, & Ścibisz, 2017; Şahin et al., 2017). Chlorophyll act as a sensitizer and facilitates photooxidation, which can increase the PV and finally may cause an unpleasant taste in oil, while carotenoid acts as a quencher and prevents oil photooxidation (Ben‐Hassine et al., 2022).

**FIGURE 2:**
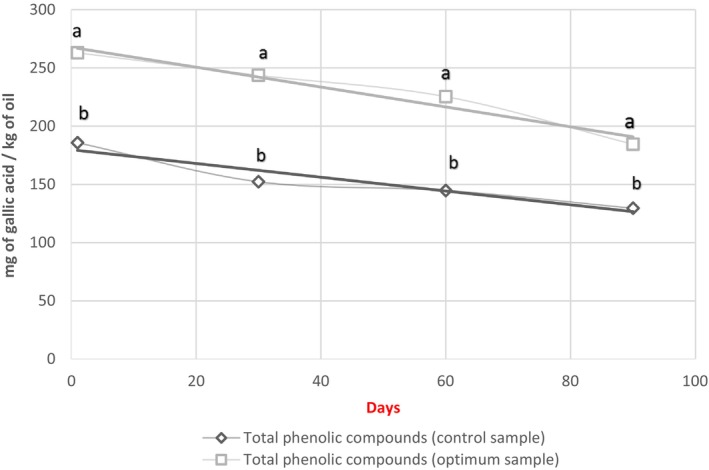
Total phenolic compounds of oil extracted from the *Nigella sativa* seeds without pre‐treatment (control sample) and with pre‐treatment in the optimum condition (optimum sample).

Results showed that OC had more chlorophyll (10.39 mg/kg) than the oil extracted from the control sample (8.56 mg/kg) (Figure 2). Microwave pre‐treatment by affecting the cell wall of BS causes destruction and therefore facilitates the release of pigments and bioactive compounds into the extracted oil. These results agreed with the previously published data by Mazaheri et al. (2019b).

Carotenoids, as a bioactive compound, can have positive effect on the prevention of many different diseases (Rowles & Erdman, 2020). The increase in the amount of carotenoids in OC was significant *(p* < .05) compared to the control sample (Figure 2). Also, the results showed that the amount of carotenoids decreased in oil samples due to their sensitivity to light, heat, and oxidation during 90 days. Destruction due to oxidation is the most important reason for reducing these compounds during storage. Carotenoids reduce oxidation in oil by deactivating sensitizers and scavenging and reducing free radicals (Mazaheri et al., 2019b). As a result, with the increase of these compounds, the peroxide value decreases, and the oxidation stability increases. Microwave pre‐treatment by heating causes denatured proteins and increases the entry of lipid‐soluble carotenoids into the oil. As a result, the presence of thermal pre‐treatments increases the bioactive compounds in the oil (Juhaimi et al., 2018). Similar results in confirmation of the obtained results showed that microwave pre‐treatment of BS and also rapeseed caused a significant increase in the total amount of chlorophyll pigments and carotenoids in the extracted oil (Ashrafi et al., 2023; Rękas, Ścibisz, et al., 2017; Rękas, Wroniak, & Ścibisz, 2017).

### 3.9 Oxidative stability

Oxidative stability of oils can be measured by different methods such as schaal test and rancimat method. Rancimat method is faster and more accurate than schaal test. Determination of oxidative stability by rancimat indicates the oils’ ability to resist oxidation by thermal treatment in the presence of air. The oxidative stability of vegetable oils is influenced by fatty acid composition, bioactive compound content, and the presence of antioxidants and proxidants (Azadmard‐Damirchi et al., 2010). Rancimat resistance of around 8 (h) is suitable as a salad oil, and 12 (h) can be used for cooking as well. Rancimat resistance above 15 (h) suits intense thermal processes such as frying (Womeni et al., 2016).

Maximum stability of BS oil was related to OC (16.5 h), and the lowest stability time (10.8 h) was related to the cold press sample without pre‐treatment (control sample). In addition, microwave pre‐treatment of BS also increased the stability of the oil samples. More oxidative stability in BS oil pre‐treated with microwave compared to the oil obtained from cold press may be due to the high amount of antioxidant compounds such as tocopherols and the increase in the amount of phenolic compounds (Mazaheri et al., 2019b). These results are similar to the findings reported by Taghvaei et al. (2014), which indicated that by increasing the microwave pre‐treatment period up to 3.5 (min), the stability of the cottonseed oil increased due to the increase in phenolic compounds. By increasing the microwave time by more than 3.5 min, the stability decreased due to increased oil oxidation. These results are in agreement with the previously published data by Bakhshabadi et al. (2018) reporting that higher oil stability can be explained by the inactivation of oxidative enzymes, such as lipase, peroxidase, and lipoxygenase.

### 3.10 Fatty acid composition

Fatty acid composition is important from nutritional and technological points of view in vegetable oils. Fatty acid composition is analyzed and determined in all edible fats and oils to monitor their changes during processing and storage. The major fatty acids present in BS oil were linoleic acid (58.04%), followed by oleic (21.9%) and palmitic acids (12.8%) (Al Juhaimi et al., 2018). Vegetable oils with high content of polyunsaturated fatty acids (PUFAs) such as black cumin seed oil with high content of linoleic acid (18:2) or flaxseed oil with high content of linolenic acid (18:3) have lower oxidative stability compared with vegetable oils with high content of saturated fatty acid (SFA) such as palm oil with high content of palmitic acid (16:0) or monounsaturated fatty acid (MUFA) such as olive oil with high content of oleic acid (18:1) (Keceli & Harp, 2014; Osman et al., 2015; Tang et al., 2021).

There were no significant differences in the fatty acid composition of oil extracted from pre‐treated BS and the control sample on the extraction day. However, linoleic acid was oxidized more in oil extracted from control BS compared with OC during the storage (Table 4). This can be explained by the higher antioxidative compounds’ content in OC compared with oil extracted from the control BS. Also, the rancimat test was approved for the lower oxidation rate in the OC, which is following the lower fatty acid oxidation rate in the OC.

**TABLE 4:** Fatty acid composition (%) of oil extracted from the *Nigella sativa* seeds without pre‐treatment (control) and with pre‐treatment in the optimum condition (optimum) during storage.

Fatty acids	Day 1		Day 90	
	Optimum*	Control	Optimum	Control
C16:0	12.90^a^ ± 0.01	13.20^a^ ± 0.01	12.22^b^ ± 0.01	13.50^b^ ± 0.01
C18:0	2.37^a^ ± 0.01	2.54^a^ ± 0.01	3.03^b^ ± 0.01	3.84^b^ ± 0.01
C18:1	23.56^a^ ± 0.01	23.68^a^ ± 0.01	24.60^b^ ± 0.01	23.08^a^ ± 0.01
C18:2	56.77^a^ ± 0.01	56.22^a^ ± 0.01	56.67^b^ ± 0.01	55.33^a^ ± 0.01
C18:3	0.55^a^ ± 0.01	0.42^a^ ± 0.01	0.41^b^ ± 0.01	0.26^b^ ± 0.01
C20:2	2.47^a^ ± 0.01	2.55^a^ ± 0.01	2.28^b^ ± 0.01	2.45^b^ ± 0.01
∑SFA	16.11^a^ ± 0.01	16.46^a^ ± 0.01	15.87^b^ ± 0.01	18.2^a^ ± 0.01
∑PUFA	59.59^a^ ± 0.01	58.99^a^ ± 0.01	59.16^b^ ± 0.01	57.93^a^ ± 0.01
MUFAs	24.0^a^ ± 0.01	24.18^a^ ± 0.01	24.97^b^ ± 0.01	23.51^b^ ± 0.01

*Note*: Different superscripts within the same line represent significant difference at (*p <* .05).

Abbreviations: MUFA monounsaturated fatty acid; PUFAs polyunsaturated fatty acids; SFA saturated fatty acid.

* pH 3, microwave time 1.25 min, and soaking level 5%.

## 4 CONCLUSION

Black cumin seed oil has high PV and AV, even in the extraction day. The main reason can be due to the high enzyme activity in BS. In this study, the effect of pH adjustment along with microwave pre‐treatment was used as a new method of BS pre‐treatment before oil extraction to control the PV and AV of extracted oil. BS was pre‐treated by soaking with a buffer system with different pHs (3 to 9) and also by a microwave with different periods of radiation (0–2.5 min). Pre‐treatment of BS by microwave and then soaking could give a higher oil extraction yield with lower PV and AV. The optimum condition for pre‐treatment of BS to extract high‐quality oil was a microwave period of 1.25 min, pH 3, and a soaking level of 5%. PV and AV of the oil extracted from the pre‐treated BS in the optimum condition were lower during storage for 90 days. Also, the bioactive components were higher in OC compared to the control oil sample. Fatty acids were well preserved in OC compared to the control oil sample. Based on the obtained results, pre‐treatment of BS with microwave and soaking with the buffers with a suitable pH can produce a relatively stable BS oil. However, there is a need for further research to control and reduce the free fatty acid formation in BS oil.

## AUTHOR CONTRIBUTIONS

Mina Sanati Agah: Investigation, Methodology, Formal analysis, Writing – original draft. Sodeif Azadmard‐Damirchi: Conceptualization, Supervision, Methodology, Review & editing. Samad Bodbodak: Supervision, Writing – original draft, review & editing.

## CONFLICT OF INTEREST STATEMENT

The author(s) declared no potential conflicts of interest with respect to the research, authorship, and/or publication of this article.

## Data Availability

The data that support the findings of this study are available from the corresponding author upon reasonable request.
